# Engineered Bacteria as Living Biosensors in Dermal Tattoos

**DOI:** 10.1002/advs.202309509

**Published:** 2024-06-17

**Authors:** Matthew E. Allen, Elina Kamilova, Carolina Monck, Francesca Ceroni, Yubing Hu, Ali K. Yetisen, Yuval Elani

**Affiliations:** ^1^ Department of Chemistry Imperial College London Molecular Sciences Research Hub London W12 0BZ UK; ^2^ Institute of Chemical Biology Imperial College London Molecular Sciences Research Hub London W12 0BZ UK; ^3^ Department of Chemical Engineering Imperial College London South Kensington London SW7 2AZ UK; ^4^ fabriCELL Imperial College London and King's College London London W12 0BZ UK

**Keywords:** bacteria, hydrogels, microfluidics, synthetic biology, tattoo, biosensors

## Abstract

Dermal tattoo biosensors are promising platforms for real‐time monitoring of biomarkers, with skin used as a diagnostic interface. Traditional tattoo sensors have utilized small molecules as biosensing elements. However, the rise of synthetic biology has enabled the potential employment of engineered bacteria as living analytical tools. Exploiting engineered bacterial sensors will allow for potentially more sensitive detection across a broad biomarker range, with advanced processing and sense/response functionalities using genetic circuits. Here, the interfacing of bacterial biosensors as living analytics in tattoos is shown. Engineered bacteria are encapsulated into micron‐scale hydrogel beads prepared through scalable microfluidics. These biosensors can sense both biochemical cues (model biomarkers) and biophysical cues (temperature changes, using RNA thermometers), with fluorescent readouts. By tattooing beads into skin models and confirming sensor activity post‐tattooing, our study establishes a foundation for integrating bacteria as living biosensing entities in tattoos.

## Graphical abstract

Tattoo based biosensors are systems that use skin as a diagnostic interface for biomarker monitoring. In this article, engineered bacteria biosensors are interfaced into dermal tattoos through the embedding of bacteria into microgels generated through microfluidics. The tattooed bacteria‐filled microgels can respond to chemical and physical cues, providing a platform that can leverage synthetic biology to design tattoo‐based biosensors.

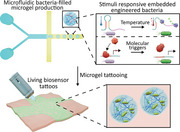

## 1 Introduction

The use of wearable diagnostic systems has blossomed over recent years through their ability to provide physiological information in real time through measurements of biomarkers in biofluids.^[^ ^1^ ^]^ In particular, tattoos have emerged as an appealing platform where skin is used as a diagnostic interface, due to their advantages of small size, wide accessibility, minimal invasiveness, aesthetic appeal, long‐term implantation, and continuous monitoring capacity.^[^ ^2^, ^3^ ^]^ Tattoos are typically present at a depth of 0.4–2.2 mm^[^ ^4^ ^]^ in the dermis, a layer of skin that contains a variety of cells, blood vessels, and nerves surrounded by interstitial fluid (ISF).^[^ ^5^, ^6^, ^7^ ^]^ Hence, tattoo‐based biosensors are a promising alternative to blood‐centered diagnostics, and have been able to sense changes in physiological biomarkers in ISF such as pH,^[^ ^8^ ^]^ metabolites,^[^ ^9^ ^]^ and electrolytes.^[^ ^10^ ^]^ Most of these systems rely on small molecule biosensors which undergo a spectral shift upon encountering the target analyte.

In synthetic biology, living cells are used as engineered biological devices. By leveraging advances in genetic engineering, cells can be transformed into engineered micromachines for therapeutics, environmental monitoring, bioremediation, chemical factories for bioproduction, and increasingly, as sensors and diagnostic agents.^[^ ^11^, ^12^ ^]^ Interest in engineered bacterial sensors, typically using detectable luminescent, fluorescent, or colorimetric signals as readouts, is increasing across multiple application domains.^[^ ^13^ ^]^ Bacteria can be designed to exhibit high analyte specificity and sensitivity using methods such as directed evolution and can be engineered to detect a broad range of molecules, including toxins, pollutants, and biomarkers.^[^ ^14^ ^]^ Bacterial sensors can also provide real‐time, continuous monitoring, crucial for dynamic biomedical settings where the environment may fluctuate over time.^[^ ^15^ ^]^ Additionally, their ability to self‐replicate and replenish their populations enables long‐term functionality without frequent maintenance.^[^ ^16^ ^]^ Engineered bacteria are also amenable to integration with biological systems, allowing them to be used to monitor biological processes, such as metabolic pathways or gene expression, and for the assessment of environmental or physiological changes.^[^ ^17^ ^]^ A final attraction is their ability to be genetically modified to enhance their sensing capabilities and detection range. Genetic circuitry and synthetic biology principles can be harnessed to integrate logic gates, advanced computational capabilities, and dynamic response profiles.^[^ ^18^ ^]^ This can also enable the connection between detection and response, such as triggering in‐situ production and secretion of biomolecular agents when specific conditions are met.^[^ ^19^ ^]^ In this context, we aim to demonstrate the feasibility of using engineered bacteria as living analytics within dermal tattoos.

To achieve this, we aimed to enclose bacteria within capsules rather than directly tattooing them as free‐floating cells, reasoning that this would enhance their functionality, stability, and safety. Capsules act as protective barriers, ensuring bacterial survival under optimal conditions, extending their lifespan, and facilitating immune system evasion.^[^ ^20^, ^21^ ^]^ Additionally, encapsulation enables precise localization, preventing bacteria from dispersing beyond the tattooed area, thereby enhancing imaging and detection capabilities, and minimizing the possibility of infection.^[^ ^22^ ^]^ Moreover, encapsulation offers protection for engineered bacteria, which is particularly pertinent when working with genetically modified strains, thus minimizing safety concerns.^[^ ^23^ ^]^

One method to ensure bacterial containment and protection is to encapsulate them within hydrogels, which are 3D porous materials that are highly hydrated, biocompatible, and mimic biological tissues^[^ ^24^, ^25^ ^]^ and which are suitable for transdermal injection.^[^ ^26^ ^]^ Within a hydrogel environment, trapped bacteria retain their activity and can interact with the small molecules that readily diffuse throughout the hydrogel matrix.^[^ ^27^, ^28^ ^]^ Hence, hydrogels are excellent materials for biosensor applications. One hydrogel that is commonly used as a foundation for bacterial biosensor devices is alginate^[^ ^29^, ^30^, ^31^, ^32^, ^33^ ^]^ which has been utilized in vivo.^[^ ^34^ ^]^ Alginate relies on the utilization of divalent ions such as Ca^2+^ to produce a 3D hydrogel architecture^[^ ^35^ ^]^ which, using microfluidic techniques, can be gelled into microscale sized beads^[^ ^36^, ^37^ ^]^ ideal for transdermal delivery. However, many hydrogel‐based bacterial biosensors have been used in bulk hydrogels, and not embedded into microscale beads, a length scale suitable for bead tattooing (smaller than tattoo needle diameters of 0.3–0.4 mm^[^ ^38^ ^]^). Instead, they have been employed as larger scale wearables^[^ ^32^, ^39^ ^]^ or used for ex vivo monitoring.^[^ ^29^, ^30^, ^40^, ^41^ ^]^

**Figure 1:**
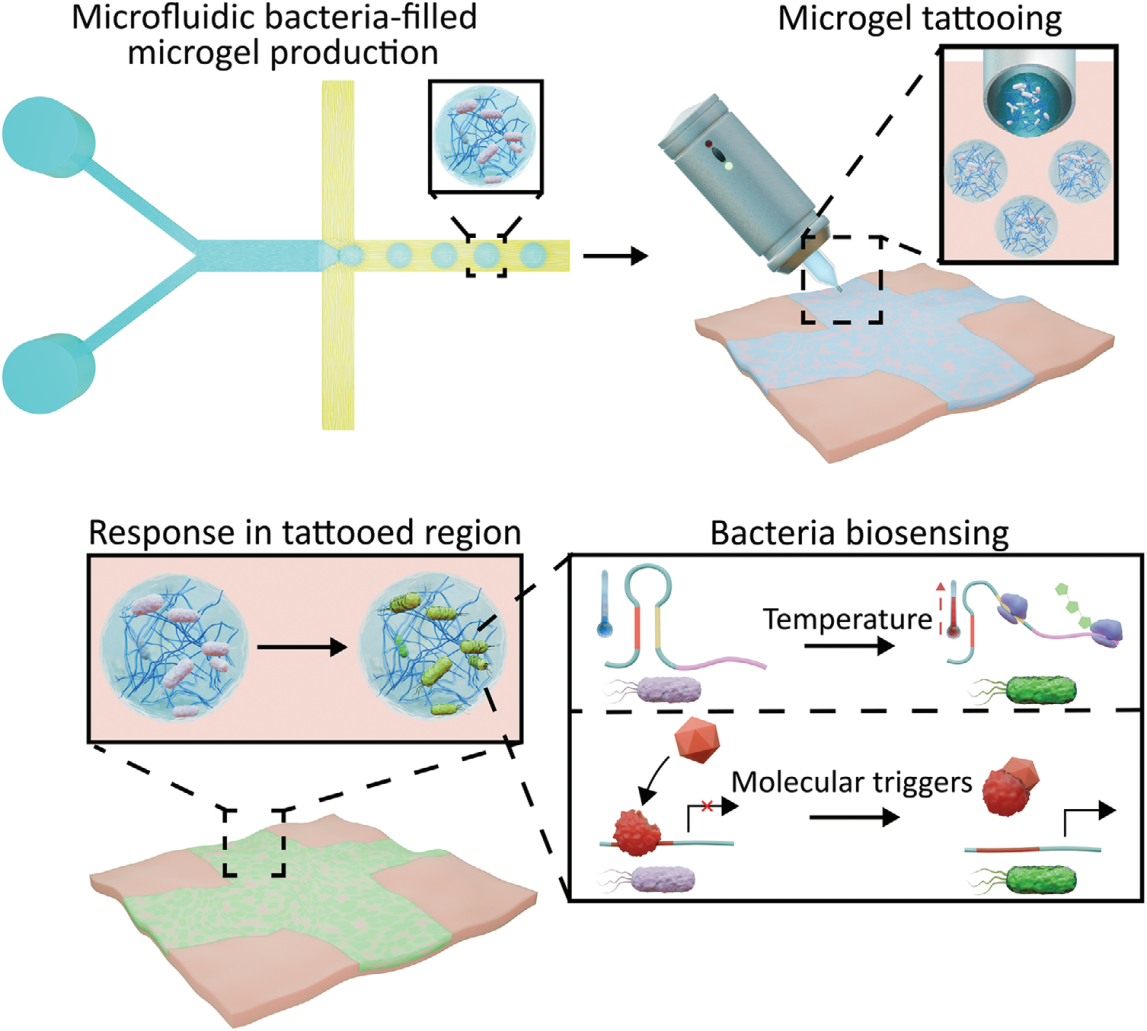
Design of dermal tattoos containing responsive bacteria‐filled microgels. Microgels containing a range of engineered bacteria with different biophysical and biochemical stimuli responses (temperature and small molecule activation) were produced using droplet microfluidics. The microgels were then tattooed into a skin mimic using a commercial tattoo gun where the embedded bacteria could respond to cues within the tattooed region.

In this proof‐of‐concept study we encapsulated *E. coli* bacterial biosensors within alginate microgels prepared through a scalable microfluidic production pipeline. We show how these biosensors can be engineered to sense both biochemical cues (model biomarkers) and biophysical ones (a change in temperature) through the production of a fluorescent signal from within the microgels. We then tattoo the bacteria‐filled microgels into a skin mimic to replicate transdermal injection. Within the skin mimic, we illustrate that the alginate microgels are tattooed in a user‐determined pattern before stimuli‐induced bacterial activation is achieved (**Figure** **1**). By demonstrating that microgels filled with bacterial biosensors can be tattooed and retain their activity post‐tattooing, we pave the way for the widescale deployment of bacteria as living biosensing agents within tattoos, potentially expanding the variety of metabolites tattoo‐based biosensors can respond to.

## 2 Results

### 2.1 Production and Characterization of Bacteria‐Filled Microgels

**Figure 2:**
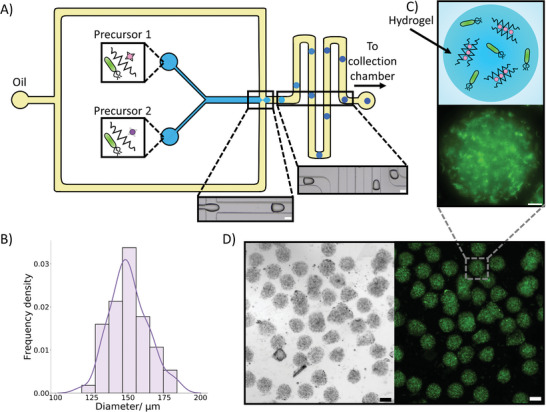
Microfluidic production and properties of bacteria‐containing microgels. A) A schematic of our microfluidic device demonstrating the production of the microgels containing bacteria. Two aqueous precursor solutions containing the bacteria meet an oil stream at a flow focusing junction where microgel precursor droplets stabilized by span 80 are formed. The precursor droplets gelate through an ion exchange reaction where the Zn^2+^ ions (purple circles) in the Zn‐EDDA complex (grey star) displace the Ca^2+^ (pink circles) from the Ca‐EDTA complex (black star). This allows the Ca^2+^ ions to crosslink the alginate (zig‐zag lines) into an egg box structure. This forms the microgel beads. The microgel beads are then collected in an external collection chamber. The brightfield images demonstrate the production of the precursor droplets and the travel of the droplets through meanders where gelation will occur. The scale bars are 100 µm. B) A histogram with a kernel density estimation fit demonstrating the diameter of the produced bacteria containing microgels. Mean diameter was 151 µm with a polydispersity index of 0.0076 (*n* = 60). C) A diagram and fluorescence image of a single bacteria‐containing microgel after bacteria expression, where the localization of fluorescent signal in the bacteria can be seen. The scale bar is 20 µm. D) Brightfield and fluorescence images of a population of bacteria‐filled microgels after bacteria protein expression. The bacteria fluorescence can be seen to be localized within the gels. The scale bars are 100 µm.

We prepared the bacteria‐containing microgel beads using a microfluidic chip (**Figure** **2**A), employing similar strategies as reported elsewhere.^[^ ^37^ ^]^ This required using a polydimethylsiloxane (PDMS) chip (Figure S1, Supporting Information) where two aqueous solutions, one containing alginate with a calcium ethylenediaminetetraacetic acid (Ca‐EDTA) complex, HEPES and *E. coli* with the other containing alginate with a zinc ethylenediamine‐N,N'‐diacetic Acid (Zn‐EDDA) complex, HEPES and *E. coli* bacteria. These aqueous streams meet an oil phase at a flow‐focussing junction. At this junction aqueous droplets containing the precursor solutions are formed and stabilized by span 80 surfactant in the oil phase. Within the produced droplets an ion exchange reaction occurred where the Zn^2+^ ions removed the Ca^2+^ ions from the EDTA complex, thus enabling the Ca^2+^ ions to crosslink the alginate and form a microgel. The ion exchange reaction was done at pH 6.4 as this pH enabled sufficiently quick enough gelation without compromising the viability of the encapsulated bacteria^[^ ^42^ ^]^ and providing homogenous encapsulation.^[^ ^36^ ^]^ The microgels were then resuspended in fresh LB media containing Ca^2+^ to be characterized with respect to their ability to respond to biophysical and biochemical cues.

We then analyzed a population of bacteria‐encapsulated microgels (Figure 2B–D). From the size distribution of the microgels (Figure 2B) it could be seen that the microfluidic device had produced microgels with a mean diameter of 151 µm and a polydispersity index of 0.0076. A value below 0.1 is considered the threshold for monodsipersity.^[^ ^43^ ^]^ Hence the microfluidic method can be considered a way to produce monodisperse microgels with encapsulated bacteria, where the size distribution can be readily altered either through adjusting the flow rates or through changing the channel dimensions.^[^ ^44^ ^]^ The concentration of the bacteria within the microgels could also be controlled by varying the quantity of bacteria inserted into the aqueous precursor solutions (Figure S2, Supporting Information). Additionally, the microscopy images in Figure 2C,D showed that the encapsulated bacteria had a localized green fluorescent protein (GFP) signal within the microgels, demonstrating that the bacteria are intact upon microgel generation. Further imaging also confirmed that the bacteria were positioned homogenously throughout the microgels (Figure S3, Supporting Information). Moreover, on a population level, the fluorescence can be seen localized to the gels, indicating limited escape of the bacteria from the microgels and high encapsulation efficiency during the microfluidic production.

### 2.2 Response of Bacteria to Stimuli within Microgels

Upon confirming that bacteria could be encapsulated within the microgels, we then demonstrated that they could be engineered to respond to both biophysical and biochemical cues. To perform these experiments, we encapsulated two different engineered bacteria strains, one carrying an RNA‐thermometer for temperature control of protein production and one carrying an Isopropyl β‐ d‐1‐thiogalactopyranoside (IPTG) inducible bio‐switch.

RNA thermometers are RNA‐based regulators of gene expression.^[^ ^45^ ^]^ They are usually placed at the 5` untranslated region of a coding gene where, at non‐permissive temperatures, they form a hairpin structure that sequesters the ribosome binding site (RBS) blocking translation. In this study, we adopted a previously designed heat‐responsive thermometer^[^ ^46^ ^]^ where temperatures above ≈35 °C provide sufficient energy to denature the secondary hairpin structure, allowing ribosomal access and subsequent translation of a protein, in our case a GFP reporter (Figure S4, Supporting Information).

**Figure 3:**
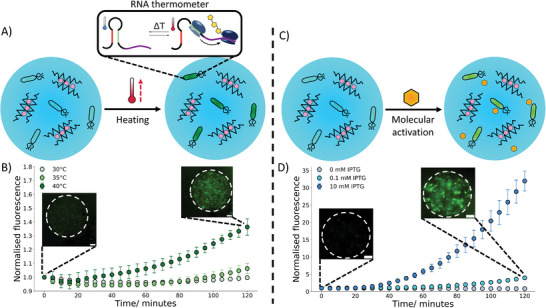
Activation of hydrogel embedded bacteria through biophysical and biochemical cues. A) A schematic demonstrating the activation of bacteria in response to temperature, upon heating a localized fluorescent signal is produced within the embedded bacteria through the expression of GFP due to the activation of an RNA thermometer. B) A 2‐hour timelapse with embedded images showing how temperature impacts the fluorescence signal of the embedded bacteria controlled by RNA thermometers. Upon heating to 40 °C where the thermometer is in an “ON” state an increase in GFP expression can be seen. At lower temperatures where the thermometer is in an “OFF” state little to no increase in fluorescence signal is seen. The microscopy images at 0 and 2 hr show the fluorescent signal from the bacteria, and the dotted lines show the hydrogel boundary. C) A diagram depicting the activation of bacteria in the presence of a chemical signal (IPTG). The IPTG enables the localized production of GFP in the bacteria. D) A 2‐hour timelapse graph with embedded microscopy images demonstrating IPTG concentration dependence at 30 °C. With an increase in IPTG concentration, a greater increase in fluorescence is observed. The microscopy images at 0 and 2 hr show the fluorescent signal from the bacteria, and the dotted lines show the hydrogel boundary. The error bars indicate 1 standard deviation from n = 10 hydrogel beads. The scale bar on all the microscopy images is 20 µm.

We first verified that the selected thermometer worked in bulk and performed differently to constitutive bacteria (Figure S5, Supporting Information). Next, we placed the bacteria within the microgels and heated them to a variety of temperatures (**Figure** **3**A,B) (Video S1, Supporting Information). It could be observed that within the 2 hour observation period at 30 °C there was a minimal increase in expression of GFP. At 35 °C, around the transition temperature of the thermometer, a similar trend was observed indicating that the thermometer was still positioned toward the OFF state. However, at 40 °C a significant increase in GFP production could be seen demonstrating that at this temperature the RNA thermometer was in a ON state. This matched the bulk experiments and showed that the properties and responses the bacteria exhibit in bulk could be transferred into a microgel successfully. At all temperatures a small decrease in GFP expression could be seen initially before a curve upwards, we have attributed this to photobleaching of the GFP. To further confirm that the properties of the bacteria were similar within the gels to in bulk we compared the GFP expression at 30 °C to that of constitutive bacteria in microgels (Figure S6, Supporting Information). We again observed that at 30 °C the constitutive bacteria expressed more GFP, matching the trends in bulk.

In an analogous fashion we then investigated incorporating bacteria that responded to a chemical trigger (IPTG) (Figure S7, Supporting Information) into the microgels (Figure 3C,D) (Video S2, Supporting Information). Here, bacterial cells are responsive to the presence of the small molecule IPTG, commonly used for induction of the lac promoter in bacterial cells.^[^ ^47^ ^]^ Again, activation of the promoter would lead to GFP production. IPTG is able to freely diffuse into the microgels and reach the bacteria as the gels are nanoporous (≈5 nm),^[^ ^48^ ^]^ a pore size larger than the size of IPTG. On IPTG activation, a dose‐dependent relationship between analyte concentration and GFP fluorescence within the microgel was seen, an important feature for a biosensor.^[^ ^49^ ^]^ These experiments were additionally conducted at 30 °C, a non‐permissive temperature for the RNA thermometer, thus expanding the capabilities of the biosensor.

Within both the temperature and IPTG‐triggered bacteria experiments, the GFP was produced intracellularly. Therefore, once activated, the bacteria signal would remain. However, the bacteria could respond again to a new cue after an initial activation by producing more GFP (Figure S8, Supporting Information). We thus demonstrate that the biosensors could dynamically sense and respond to the surrounding environment, an important property for a biosensor.

Finally, we assessed the stability of the microgels and the viability of the bacteria within by observing them over extended periods of time. It could be seen that in 12 hours at room temperature (Video S3, Supporting Information), the bacteria within the microgels grew into microcolonies,^[^ ^50^ ^]^ with limited bacterial escape observed. The bacteria that had grown in microcolonies within the microgels additionally responded to the addition of IPTG after 20 hours, demonstrating extended viability (Figure S9, Supporting Information). However, on leaving for 7 days in buffer (Figure S10, Supporting Information), the microcolonies did not exhibit activity after IPTG addition, although the microgels remained intact. Our results show that the microgels were stable for the lifetime of the bacteria and therefore an appropriate containment unit.

### 2.3 Tattooing Bacteria Embedded Microgels

**Figure 4:**
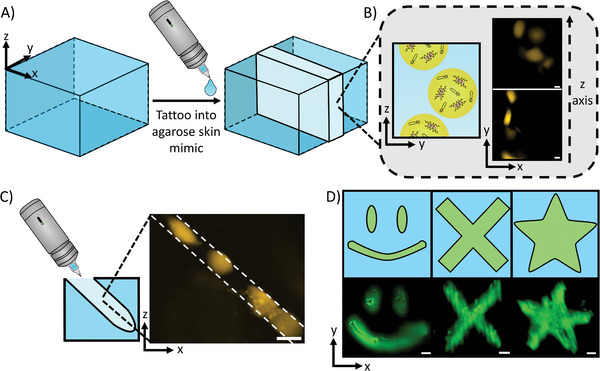
Tattooing of bacteria‐filled microgels into agarose skin mimics. A) A schematic demonstrating the tattooing of an agarose hydrogel block. Upon using the tattoo gun, a region of the gel (shown by the lighter blue) was filled with the material placed within the tattoo gun. B) A diagram with accompanying microscopy images showing the tattooing of bacteria‐filled microgels into the agarose block. Imaging through the tattooed region revealed that microgels were present at a range of different heights. The scale bars are 50 µm. C) A schematic with a fluorescence microscopy image showing an injection profile from the tattoo gun. The tattooed gels are distributed in a line along where the tattoo gun had made an incision in the agarose gel. The scale bar is 100 µm. D) Fluorescence images and drawings of the various shapes made by tattooing the agarose block. A smiley face, cross, and star could be tattooed into the block readily, demonstrating the versatility of shapes that can be readily produced from tattooing. The scale bars are 500 µm.

After demonstrating that bacteria embedded in the microgels could respond to biochemical and biophysical cues in the same manner as in bulk, we then sought to tattoo the microgels into an agarose skin mimic. Agarose was chosen for its transparency, which facilitates imaging, its controllable properties, and its established use in mimicking skin.^[^ ^51^, ^52^, ^53^ ^]^ To tattoo the microgels into the skin mimic, we loaded the microgel containing solution into a commercial tattoo gun (Figure S11, Supporting Information). The tattoo gun worked by piercing the agarose mimic with a microscale needle array. Upon the loaded tattoo gun piercing the skin mimic, the bacteria‐filled microgels were released from the needle and positioned within the site of piercing. To ensure that the microgels could survive being ejected from the tattoo gun, we first tattooed them into a buffer solution (Figure S12, Supporting Information), we could see that the gels retained their shape and still encapsulated bacteria, thus confirming that microgels are a viable material for tattooing. We then tattooed the gels into the skin mimic vertically for lining tattooing (**Figure** **4**A) (Figure S13, Supporting Information). For these experiments we additionally tagged the microgels with fluorescent alginate to enable increased visibility; the addition of this fluorescent alginate dye had no impact on the visibility of the bacteria signal due to minimal spectral overlap (Figure S14, Supporting Information). Upon tattooing the microgels into the skin mimic a Z stack was taken through the tattooed region (Video S4, Supporting Information; Figure 4B). Throughout the Z stack we could see the microgels positioned at a variety of depths from 0 to 1.64 mm, an indication that the tattoo gun was embedding the gels within the skin mimic and illustrating the ability to place the microgels in different skin layers, thus opening up the ability to sense and respond across multiple layers of skin.^[^ ^54^ ^]^ The Z stack was also performed through a typical depth of tattooing (≈1.5 mm^[^ ^4^ ^]^), further showing the ease that the microgels could be tattooed. In comparison, tattooing unencapsulated bacteria into the agarose mimic showed poor positioning of unprotected bacteria, each cell possessing a weak fluorescent signal, which was highly diffuse and appeared throughout the tattooed region (Figure S15, Supporting Information). This demonstrates that the tattooed bacteria filled microgels offer protection to the encapsulated bacteria and provide a more prominent localized signal.

To further confirm that the microgels were being tattooed into the skin mimic, we sliced the skin mimic and positioned it on its side, this enabled us to see holes being created by the tattoo gun needle (Figure 4C). We could see that the tattooed microgels were arranged in a line in the holes indicating that the tattoo gun is successfully tattooing the microgels into the skin mimic at an angle of 45° for shading tattooing. Hence, the depth and alignment of microgels can be adjusted by the tattooing process to be injected at precise positions. Moreover, the gels positioned within the tattoo line and within the Z stack (Figure 4B) appeared less spherical than the microgels in buffer indicating that the microgels are more constrained within the skin mimic, once again indicating that they are being positioned by the tattoo gun. We also observed that within the tattoo, the bacteria could grow within the tattooed microgels (Figure S16, Supporting Information) and the tattooed microgels could hold the bacteria for extended periods of time (Figure S17, Supporting Information), matching the results from in buffer, showing that the functionality from in buffer is transferred into the tattooed system.

To demonstrate the versatility of the tattoo platform, we loaded the tattoo gun with 0.01 mM of 500 KDa fluorescent dextran in the microgel buffer. This enabled us to readily visualize the tattoos because of the low diffusivity of the large dextran macromolecule within the skin mimic,^[^ ^55^ ^]^ allowing the dextran to remain in the tattooed regions in the time period required for observation. Through this we tattooed a variety of shapes into the skin mimic and observed them with fluorescence microscopy (Figure 4D).

Finally, to show that the bacteria‐filled microgels could be tattooed into skin and not just a skin mimic, we encapsulated tattoo ink into the microgels (Figure S18, Supporting Information) and tattooed the microgels into porcine skin, a mimic similar to human skin^[^ ^56^ ^]^ (Figure S19, Supporting Information). It could be seen that the tattoo ink‐filled microgels were present in a region of porcine skin, with evidence of the tattoo ink remaining in the microgels. These results show that the microgels are appropriate for tattooing into skin, and that our platform can be used for cosmetically appealing tattoo designs. This will maximize the deployment of such sensors for public use, a key consideration for biosensors.

### 2.4 Response of Bacteria‐Containing Tattoos to Chemical Cues

**Figure 5:**
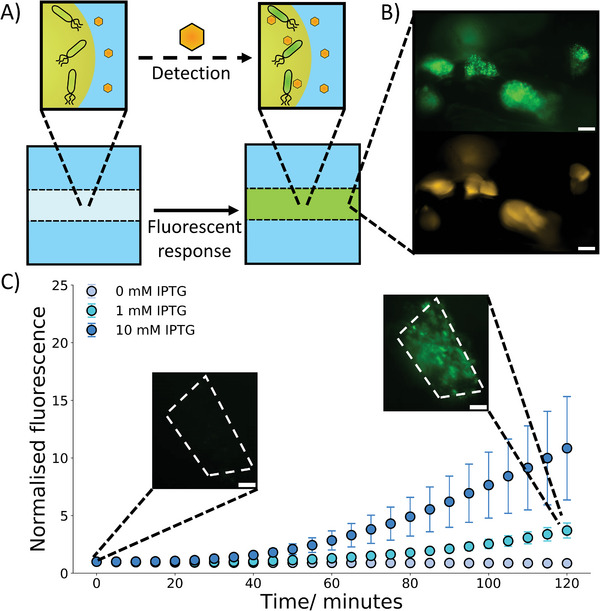
Response of tattooed bacteria‐filled microgels to chemical cues. A) A drawing showing the response of tattooed bacteria‐filled microgels to an external chemical cue (IPTG). Upon detection of the IPTG molecule the bacteria will start to produce GFP and a fluorescent signal within the tattoo. B) Microscopy images of tattooed microgels after 4 hours of bacterial activation with 1 mM IPTG in the agarose block at 30 °C. GFP signal can be observed in the same location as the microgels showing that the bacteria have sensed and responded to the IPTG within the agarose gel. The scale bars are 100 µm C) A 2‐hour timelapse graph with embedded microscopy images demonstrating the response of the tattooed microgels to external IPTG at 30 °C. With IPTG being present in the external hydrogel an increase in fluorescence signal from the bacteria is observed and shows a concentration dependent response. This is not observed when no IPTG is present. The microscopy images at 0 and 2 hours show the fluorescent signal from the bacteria evolving over time in response to 1 mM IPTG detection. The dotted lines show the position of the tattooed microgel the bacteria are embedded within. The error bars indicate 1 standard deviation from *n* = 10 tattooed hydrogel beads. The scale bar is 20 µm.

After determining that the bacteria‐filled microgels could be tattooed into a skin mimic, we then sought to demonstrate that the bacteria could respond to a stimulus when tattooed, in the same manner as in bulk, and when the microgels were present in buffer. To perform this experiment, we included the model biomarker IPTG in the skin mimic and tattooed into it a population of microgels containing bacteria that would respond to IPTG. Hence, when the bacteria‐filled microgels were tattooed into the skin mimic, they would be able to detect the IPTG present and respond by expressing GFP (**Figure** **5**A) due to the IPTG diffusing into the microgels. 1 and 10 mM IPTG was used as this would provide a clear change in fluorescent signal over the analyzed time period whilst being a physiologically relevant concentration for ISF metabolites.^[^ ^57^ ^]^ We also ensured that the tattooed agarose skin mimics were surrounded by LB buffer containing the required amount of IPTG, to keep the concentration of the IPTG in the skin mimic constant.

After incubation for 4 hours at 30 °C the tattoos were imaged (Figure 5B). It could be seen that in the regions where the tattooed microgels were placed (yellow fluorescence channel) there was also a corresponding green fluorescence from the bacteria producing GFP. This shows that the bacteria are both contained and protected within the tattooed microgels after 4 h and are producing an interpretable signal from within the tattoos.

To further show that the bacteria‐filled microgels are responding to the chemical cue upon being tattooed a 2‐hour timelapse was recorded (Figure 5C) (Video S5, Supporting Information). When the skin mimic contains IPTG an increase in fluorescence intensity can be seen, indicating that the IPTG is diffusing into the tattooed microgels and being sensed by the bacteria. The IPTG also showed a concentration dependent response, mirroring the results observed in bulk. Without IPTG no response is seen from the tattoos. These experiments confirm that as well as being able to be tattooed easily, the microgels also sense and respond to chemical signals in the same dose dependent manner as in bulk and as microgels in buffer. Therefore, rapid prototyping and optimization of the bacterial biosensors in bulk is shown,^[^ ^58^ ^]^ saving significant costs and time before the bacteria can be placed in the microgels and tattoos and perform an identical function, which is ideal for the development of complex biosensors.

## 3 Discussion

Our system is underpinned by microfluidic production of bacteria‐filled microgels, which enables continuous creation of the tattoo precursors. This process is higher throughput than batch methods used to make hydrogels^[^ ^59^ ^]^ and thus is vital for the scale up of the tattoo platform to reach widespread utilization across society. This is further supported by our use of standard user‐friendly tattoo equipment and procedures available in most tattoo studios. Furthermore, microfluidics enables the possibility of utilizing different populations of microgel‐containing bacteria in one system, allowing for the simultaneous sensing and detection of multiple stimuli independently. Our work also opens up the possibility of generating different bacteria‐filled microgels populations, each sensing a different stimulus, which could be considered as tattoo “inks”. The “inks” are biocompatible and can sense a wider range of stimuli for longer periods of time compared to small molecule‐based biosensors.^[^ ^60^ ^]^ These “inks” could be inserted into the same tattooed region or in different regions providing the ability to sense multiple stimuli in one area or multiple stimuli in different areas. The versatility and biocompatibility of the alginate building blocks could also be leveraged in future work, for example by incorporating hydrogel degradation enzymes into our microgels, leading to a naturally degradable tattoo.^[^ ^61^ ^]^

Moreover, the tattoo biosensors can be altered on the bacterial level, by changing the bacteria's genetic circuitry, which can be further enhanced to incorporate logic gates and more advanced biocomputational elements,^[^ ^62^ ^]^ as well as systems that enable tighter gene expression control.^[^ ^63^ ^]^ This is relevant considering that logic gates are hard to replicate within conventional tattoo biosensor platforms and are required for monitoring complex biological systems, where the detection of a combination of analytes is required to give a descriptive readout.^[^ ^64^ ^]^ The modular functionality of the microgels will also enable the incorporation of non‐living sensors into tattoos, allowing the creation of a biohybrid tattoos^[^ ^65^ ^]^ and further increasing the scope of stimuli that could be detected. Thus, these methods enable increased customizability of the tattoo platform, which is vital for creating a broadly utilized biosensor system that could be applied to monitor health through biomarker detection^[^ ^66^ ^]^ in ISF, provide a triggerable response to infections^[^ ^67^ ^]^ and selectively mark regions of skin,^[^ ^68^ ^]^ both for cosmetic purposes and for targeted therapies.

## 4 Conclusions

In conclusion, we have developed a bacteria biosensing platform that can be readily utilized in a tattoo‐based system as living analytics. We demonstrate a microfluidic pipeline for high‐throughput production of bacteria‐filled microgels, whereby the encapsulated bacteria can respond to both biophysical and biochemical cues through the utilization of RNA thermometers and detection of model biomarkers. We then show that these microgel populations can be utilized as “inks” for tattooing into a hydrogel skin mimic at a controllable depth and angle. Upon tattooing, the bacteria remain enclosed by the microgels (preventing escape and associated biomedical consequences) and can respond to biochemical signals in the same manner as the buffered solutions. Our strategy for tattoo production is scalable by exploiting microfluidic technologies and the tattoos are simple to generate using commercial equipment. It also provides the ability to readily interchange the bacteria within the microgels enabling the usage of a vast array of bacterial,^[^ ^69^, ^70^ ^]^ and possibly mammalian,^[^ ^71^ ^]^ biosensors for detection, similarly to what was recently shown for freeze‐dried, cell‐free synthetic circuits.^[^ ^72^ ^]^ Compared to these, our system leverages the ability of bacterial cells to operate more complex circuits with sustained responses over time. We anticipate that this initial proof‐of‐concept demonstration will help usher in the widescale deployment of synthetic biology within tattoo‐based biosensors and produce next generation biosensors for use in both research and within society.

## 5 Experimental Section

### Materials

Sodium alginate was obtained from Sigma Aldrich (Gillingham, UK) and used without further purification. Ethylenediamine‐N,N'‐diacetic Acid (EDDA) was purchased from Tokyo Chemical Industry. Rhodamine B labeled alginate was purchased from Haworks (New Jersey, USA). Panthera black liner tattoo ink was purchased from Wanda tattoo (London, UK). The porcine skin tissue was obtained from a local supermarket (London, UK). All other reagents that include, Calcium Chloride, Zinc Acetate, Ethylenediaminetetraacetic acid (EDTA), Agarose, HEPES buffer, span 80, mineral oil, Isopropyl β‐D‐1‐thiogalactopyranoside (IPTG), 500 KDa Fluorescein isothiocyanate–dextran, Ampicillin and Luria Broth (LB) were all purchased from Sigma Aldrich (Gillingham, UK) unless specified.

### Microfluidic Device Manufacture

The silicon master wafers (Inseto) used to produce the microfluidic devices were made by depositing a photoresist (SU‐8 3050, Kayaku Advanced Materials, MA, USA) of 100 µm depth using a spin coater. The wafers were then baked before UV light exposure (365 nm, 300 mJ cm^−2^) through an acetate photomask (Micro Lithography services, UK) which had the device design on. After a post exposure bake, the unexposed features were eliminated using propylene glycol monomethyl ether acetate developer and rinsed with Isopropyl alcohol. The patterned wafers were silanised with trichloro(1H, 1H, 2H, 2H‐perfluorooctyl)silane under a vacuum overnight.

The patterned wafers then had degassed Polydimethylsiloxane (PDMS) Sylgard 184 elastomer (10:1 Elastomer: Curing agent) obtained from Dow Corning (Michigan, USA) poured onto them before at least 3 hours of curing at 60 °C. The patterned PDMS was then removed from the underlying wafer before 1.5 mm holes were punched into the PDMS for the inlet and outlet ports. The PDMS was then irreversibly bonded to a glass slide in order to seal the microfluidic channels through exposing both the glass slide and patterned side of the PDMS to plasma (Harrick Plasma, NY, USA) for 90 seconds before contacting the surfaces together. The devices were then left overnight before use to ensure complete bonding between the glass slide and PDMS.

### Preperation of Bacteria

A plasmid containing dasher GFP under a T7 promoter was synthesized by DNA 2.0 (CA, USA), comprising an *E. coli* pJexpress441 vector with T7 promoter and terminator sequences (Catalogue number: FP‐03‐441) (Figure S20, Supporting Information). BL21‐DE3 *E. coli* containing IPTG‐inducible T7 polymerase was used for the expression of GFP under T7 control.

For the temperature‐responsive expression, a plasmid containing dasherGFP under the control of an RNA thermometer sequence was used (Figure S21, Supporting Information). This plasmid was expressed in DH5α *E. coli*.

An overnight culture was prepared from a plated colony grown in 4 mL LB with 100 µg mL^−1^ Ampicillin at 30 °C, 250 rpm. The following day, tubes were prepared containing 40 µL of overnight culture and 2 mL of fresh LB with 100 µg mL^−1^ of Ampicillin. These were incubated at 30 °C at 250 rpm for 1 h, or until the bacteria reached the exponential growth phase.

For use in the microgels, upon reaching the exponential phase, the 2 mL of renewed bacterial culture was centrifuged for 5 min at 3000 x g and the supernatant was discarded. This produced a pellet ready to be rehydrated by the aqueous alginate solutions.

### Alginate Microgel Production

Stock solutions of 2 wt.% alginate, 84 mM Ca‐EDTA or 84 mM Zn‐EDDA, and 40 mM HEPES (pH 6.4) were made up. These stock solutions were diluted by a factor of 2 in deionized water and then used to resuspend the bacterial pellet. This gave final aqueous solutions containing 1 wt.% alginate, 42 mM Ca‐EDTA or 42 mM Zn‐EDDA, 20 mM HEPES (pH 6.4) and *E. coli* (BL21‐DE3, DH5α strains). The oil phase solution was prepared through dissolving 5 wt.% Span 80 into mineral oil. For experiments including the rhodamine labelled alginate the fluorescent alginate was added at 0.1 wt.% to the alginate precursor solutions. Aqueous phases containing the tattoo had 1 wt.% alginate, 42 mM Ca‐EDTA or 42 mM Zn‐EDDA, 20 mM HEPES (pH 6.4), and the tattoo ink diluted fourfold from the stock.

The aqueous and oil phases were then flowed through the appropriate inlets of the PDMS microfluidic device (Figure S1, Supporting Information). Polyethylene tubing (Kinesis, UK) was used to connect the solution reservoirs both to the microfluidic device and a pressure pump (Elveflow, Paris, France) was utilized to control the flow rates of the aqueous and oil phases. The aqueous flow rates were adjusted to ensure an equal distribution of both aqueous phases were flowing through the device throughout the bead production. The oil phase flow rate was always larger than that of the aqueous flow rates to ensure aqueous droplet formation occurred at the aqueous/ oil interface.

The produced hydrogel beads were collected by outlet tubing from the microfluidic device connecting to an Eppendorf tube. These collected beads were centrifuged for 2 min at 2600 x g (Camlab D1008) to produce a pellet. The oil phase supernatant was then removed before the pellet was resuspended in LB containing 20 mm Ca^2+^. The additional calcium in the LB prevented the unwanted degradation of the alginate beads from occurring. In the case of hydrogel beads containing tattoo ink, 5 centrifugation, and resuspension cycles were done to remove any unencapsulated ink from the aqueous solution.

The polydispersity index (PDI) of the bead size distribution was calculated using Equation 1 where σ is the standard deviation of the distribution and *d* is the mean.^[^ ^73^ ^]^

(1) $$PDI\;={\left(\frac{\sigma }{d}\right)}^{2}$$

### Tattooing Alginate Microgels into Agarose Skin Mimics

1 wt.% of agarose was dissolved in boiling LB with 20 mM Ca^2+^ before being left to cool in a PDMS mould at room temperature. This produced an agarose block upon which to tattoo into. For experiments containing IPTG in the agarose block, 1 mM of IPTG was added to the boiling LB solution.

A Cheyenne sol nova unlimited 4.0 tattoo gun with a Cheyenne capillary cartridge 3‐Liner was used to perform the tattooing experiments into the agarose blocks. The diameter of the needle was 0.3 mm which is larger than the size of the ejected microgels. The tattoo gun was loaded with 0.01 mM 500 KDa Fluorescein isothiocyanate–dextran and 20 mM Ca^2+^ in LB to demonstrate the ability to tattoo into the agarose blocks. For tattooing the gels into the agarose blocks the tattoo gun was loaded with alginate microgels in LB containing 20 mM Ca^2+^. For tattooing the bacteria into the agarose blocks the tattoo gun was loaded with bacteria in LB containing 20 mM Ca^2+^.

For imaging the tattoo gun tattooing into the agarose block (Figure S11, Supporting Information) an iPhone 13 camera was used.

### Tattooing Alginate Microgels into Porcine Skin

A Cheyenne sol nova unlimited 4.0 tattoo gun with a Cheyenne capillary cartridge 3‐Liner was used again to perform the tattooing experiments into the porcine skin. For tattooing the microgels into the porcine skin, the tattoo gun was loaded with alginate microgels in LB containing tattoo ink.

Imaging the tattooed region in color was performed by using an iPhone 13 camera capturing through a GXM‐XTL‐201 stereoscope (GT Vision).

### Microfluidic Microscopy

A Nikon eclipse Ts2R microscope with a Nikon DS‐Fi3 camera and a 4x objective was used to image the microfluidic chip for the production of the bacteria containing alginate beads.

### Plate Reader Bacteria Analysis

Validation of the stimuli‐responsive systems was conducted using a plate reader to monitor dasher GFP expression through recorded fluorescent signal. 100 µL of temperature‐responsive culture was transferred to a Tecan Infinite M NANO+ plate reader. For the IPTG responsive strain, the cultures were incubated at 30 °C for 8 hours, measuring optical density (OD, wavelength = 600 nm) and fluorescence (Ex: 495, Em: 528) every 20 min. For the RNA thermometer and constitutive strains, the cultures were incubated for 1 day with a variable incubation temperature (30, 34, 38, and 42 °C), measuring optical density (OD, wavelength = 600 nm) and fluorescence (Ex: 495, Em: 528) every 30 min. For both experiments shaking incubation was set between reads and was paused 20s before every measurement. For the RNA thermometer comparison (Figure S5, Supporting Information), endpoint fluorescence readings were normalized with respect to optical density (OD) (Equation 2) before being divided by the normalized fluorescence at 30 °C ($F_{30^{\circ }C}$) (Equation 3) to calculate the fold change.

(2) $$OD\;normalised\;fluorescence\;=\;\frac{F_{t}}{OD}$$

(3) $$Fold\;change\;=\;\frac{OD\;normalised\;fluorescence}{F_{30^{\circ }C}}$$

For the IPTG bacteria colony response curves, the fluorescence was normalized by first normalizing with respect to optical density and then normalizing to the initial fluorescence (*F*_0_) (Equation 4).

(4) $$Normalised\;fluorescence\;=\;\frac{\left(F_{t}/OD_{t}\right)}{\left(F_{0}/OD_{0}\right)}$$

### Timelapse Microscopy

A Nikon eclipse Ti2‐E inverted microscope with a D‐LEDI and a Prime BSI express camera was used to image the populations of bacteria containing alginate beads and performed the timelapse experiments involving the beads and agarose containing tattooed beads. The solutions or agarose blocks were placed in PDMS wells on a cover slip, another cover slip was placed on top to seal the sample chambers. Fluorescence imaging was done with a FITC filter cube to image fluorescein tagged dextran and GFP. A TRITIC filter cube was used to image the rhodamine labelled gels. For heating a Linkam PE100 Peltier heating stage with a T96 controller and a water circulation pump was placed on the microscope and the samples remained at their desired temperature (30, 35, or 40 °C) for the duration of the experiments. For the microscopy images obtained in Figure 1, the bacteria filled microgels were induced with 10 m IPTG for 4 hours at 30 °C. For the timelapse experiments, the normalized fluorescence intensity was obtained through Equation 5 where *F_t_* is the fluorescence intensity at a time point and *F*_0_ is the initial fluorescence intensity.

(5) $$Normalised\;fluorescence\;=\;\frac{F_{t}}{F_{0}}$$

## Conflict of Interest

The authors declare no conflict of interest.

## Author Contributions

M.E.A. and E.K. contributed equally to this work. M.E.A. designed and performed the experiments, analyzed the data, and wrote the manuscript. E.K. performed experiments and analyzed data. C.M. designed and performed experiments and analyzed data. F.C. helped revise the manuscript. A.Y. designed experiments and helped revise the manuscript. Y.H. designed experiments and helped revise the manuscript. Y.E. designed experiments, revised the manuscript, and supervised the project as a whole.

## Supporting information

Supporting Information

Supplemental Video 1

Supplemental Video 2

Supplemental Video 3

Supplemental Video 4

Supplemental Video 5

## Data Availability

The data that support the findings of this study are available from the corresponding author upon reasonable request.
