# Supplementary material for: Engineered Bacteria as Living Biosensors in Dermal Tattoos
Source: Adv Sci (Weinh). 2024 Jun 17;11(30):2309509. doi: 10.1002/advs.202309509 (PMC11321667; doi:10.1002/advs.202309509)
Supplement: Supplementary file 1 — Supporting Information [file ADVS-11-2309509-s001.pdf]

ADVANCED SCIENCE
Open Access

Supporting Information

for *Adv. Sci.*, DOI 10.1002/advs.202309509

Engineered Bacteria as Living Biosensors in Dermal Tattoos

Matthew E. Allen, Elina Kamilova, Carolina Monck, Francesca Ceroni, Yubing Hu, Ali K. Yetisen and Yuval Elani**

Supporting Information

Engineered Bacteria as Living Biosensors in Dermal Tattoos

Matthew E. Allen[1,2,3,4†], Elina Kamilova[3†], Carolina Monck[3], Francesca Ceroni[3], Yubing Hu[3], Ali K. Yetisen[3], Yuval Elani[2,3,4*]*

[1]Department of Chemistry, Imperial College London, Molecular Sciences Research Hub, London, W12 0BZ, UK

[2]Institute of Chemical Biology, Imperial College London, Molecular Sciences Research Hub, London, W12 0BZ, UK

[3]Department of Chemical Engineering, Imperial College London, South Kensington, London SW7 2AZ, UK

[4]fabriCELL, Imperial College London and King's College London, London, W12 0BZ, UK

† These authors contributed equally

E-mail: yubing.hu@imperial.ac.uk; y.elani@imperial.ac.uk

Supporting figures and tables

**Bacteria strain**	**Depicted in figures**
IPTG responsive BL21-DE3 *E. coli*	Figures 2, 3 and 5<br>SI figures 2, 3, 7, 8, 9, 10, 12, 15, 16 and 17<br>Videos S2, S3 and S5
DH5α *E. coli* containing RNA thermometers	Figures 3 and 4<br>SI figures 5, 6, 13 and 14<br>Videos S1 and S4
DH5α *E. coli* without RNA thermometers	SI figures 5 and 6

Table S1: Bacteria strains used within the different figures throughout the manuscript.

Plasmid	Sequence
LacO – T7 – dasherGFP	GGAATTGTGAGCGGATAACAATTCCCCTCTAGAAATAATTTTG<br>TTTAACTTTTTAATACGACTCACTATAGGGGAATTGTGAGCGGA<br>TAACAATTCCCCTCTAGAAATAATTTTGTTTAACTTTTAGGAGG<br>TAAAAATGACGGCATTGACGGAAGGTGCAAAACTGTTTGAGAA<br>AGAGATCCCGTATATCACCGAACTGGAAGGCGACGTCGAAGGT<br>ATGAAATTTATCATTAAAGGCGAGGGTACCGGTGACGCGACCA<br>CGGGTACCATTAAAGCGAAATACATCTGCACTACGGGCGACCT<br>GCCGGTCCCGTGGGCAACCCTGGTGAGCACCCTGAGCTACGGT<br>GTTCAGTGTTTCGCCAAGTACCCGAGCCACATCAAGGATTTCTT<br>TAAGAGCGCCATGCCGGAAGGTTATACCCAAGAGCGTACCATC<br>AGCTTCGAAGGCGACGGCGTGTACAAGACGCGTGCTATGGTTA<br>CCTACGAACGCGGTTCTATCTACAATCGTGTCACGCTGACTGGT<br>GAGAACTTTAAGAAAGACGGTCACATTCTGCGTAAGAACGTTG<br>CATTCCAATGCCCGCCAAGCATTCTGTATATTCTGCCTGACACC<br>GTTAACAATGGCATCCGCGTTGAGTTCAACCAGGCGTACGATA<br>TTGAAGGTGTGACCGAAAAACTGGTTACCAAATGCAGCCAAAT<br>GAATCGTCCGTTGGCGGGCTCCGCGGCAGTGCATATCCCGCGT<br>TATCATCACATTACCTACCACACCAAACTGAGCAAAGACCGCG<br>ACGAGCGCCGTGATCACATGTGTCTGGTAGAGGTCGTGAAAGC<br>GGTTGATCTGGACACGTATCAGTGA
J23118b – RNAT3-2 – dasherGFP	TTGACGGCTAGCTCAGTCCTAGGTATTGTGCTAGCATAGAGCTT<br>TTTAAAAAAAAAAAGTACTAAGGAGTACTAGATGACGGCATTG<br>ACGGAAGGTGCAAAACTGTTTGAGAAAGAGATCCCGTATATCA<br>CCGAACTGGAAGGCGACGTCGAAGGTATGAAATTTATCATTAA<br>AGGCGAGGGTACCGGTGACGCGACCACGGGTACCATTAAAGC<br>GAAATACATCTGCACTACGGGCGACCTGCCGGTCCCGTGGGCA<br>ACCCTGGTGAGCACCCTGAGCTACGGTGTTCAGTGTTTCGCCA<br>AGTACCCGAGCCACATCAAGGATTTCTTTAAGAGCGCCATGCC<br>GGAAGGTTATACCCAAGAGCGTACCATCAGCTTCGAAGGCGAC<br>GGCGTGTACAAGACGCGTGCTATGGTTACCTACGAACGCGGTT<br>CTATCTACAATCGTGTCACGCTGACTGGTGAGAACTTTAAGAA<br>AGACGGTCACATTCTGCGTAAGAACGTTGCATTCCAATGCCCG<br>CCAAGCATTCTGTATATTCTGCCTGACACCGTTAACAATGGCAT<br>CCGCGTTGAGTTCAACCAGGCGTACGATATTGAAGGTGTGACC<br>GAAAAACTGGTTACCAAATGCAGCCAAATGAATCGTCCGTTGG<br>CGGGCTCCGCGGCAGTGCATATCCCGCGTTATCATCACATTAC<br>CTACCACACCAAACTGAGCAAAGACCGCGACGAGCGCCGTGA<br>TCACATGTGTCTGGTAGAGGTCGTGAAAGCGGTTGATCTGGAC<br>ACGTATCAGTGA

Table S2: Sequences of the relevant parts of all plasmids used in this work. The colours on the plasmid names correspond to the relevant parts of the sequence.

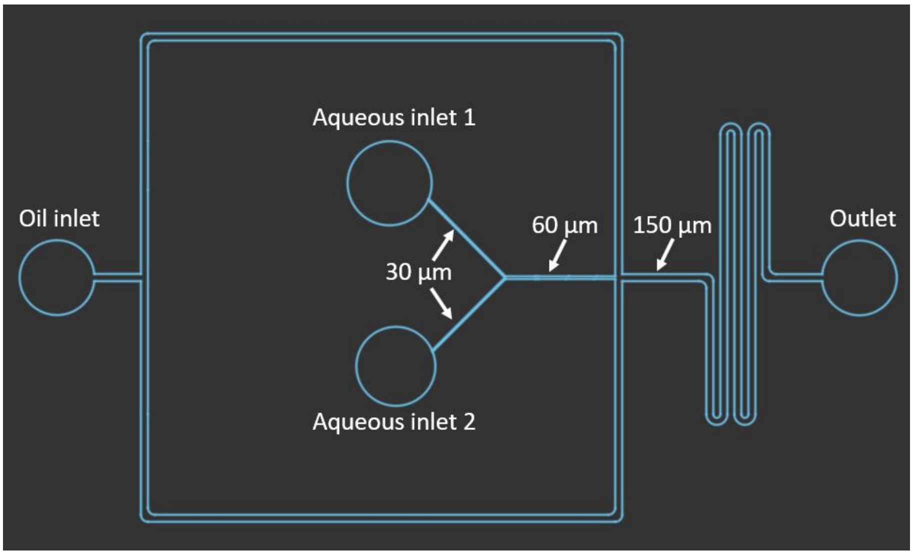

Figure S1: Technical schematic of the microfluidic device used. The different channel widths and inlets/outlets are annotated within the schematic. The channel depth was always 100 µm.

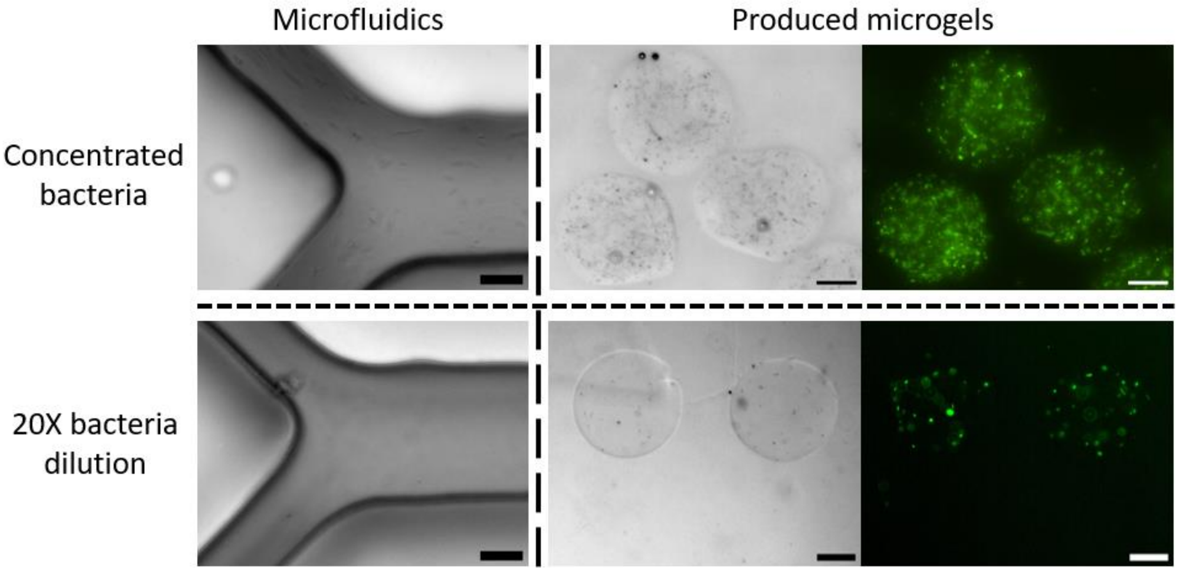

Figure S2: Variation of bacteria concentration in microgels. Brightfield images of the aqueous Y junction in the microfluidic device showing two different bacteria concentrations being used to form microgel populations. The dilute bacteria concentration is a 20X dilution of the concentrated solution. The brightfield and fluorescence images of the produced microgels show different concentrations of bacteria embedded within. The different bacteria concentrations in the microgels mirror the concentrations present in the microfluidic device, thereby demonstrating that the bacteria concentration in the microgels can be controlled. The scale bars are 20 µm for the microfluidic device images and 50 µm for all the microgel images.

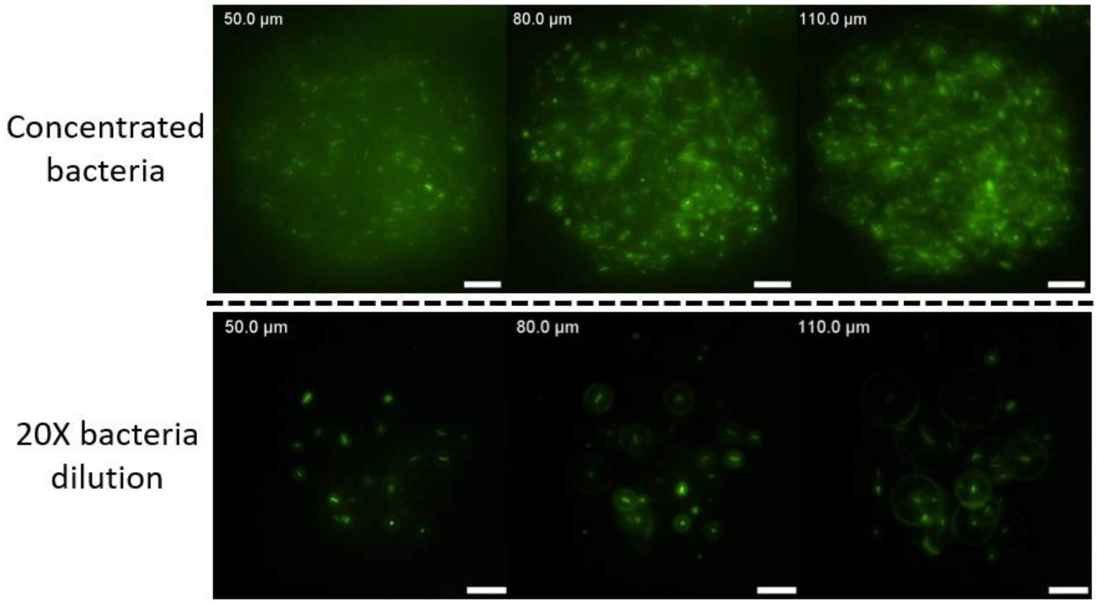

Figure S3: Z stacks of bacteria encapsulated in microgels. Fluorescence images of two bacteria filled microgels at a variety of heights. It can be seen that different bacteria are present in each plane, indicating that the bacteria are encapsulated throughout the produced microgels. The scale bars are 20 µm.

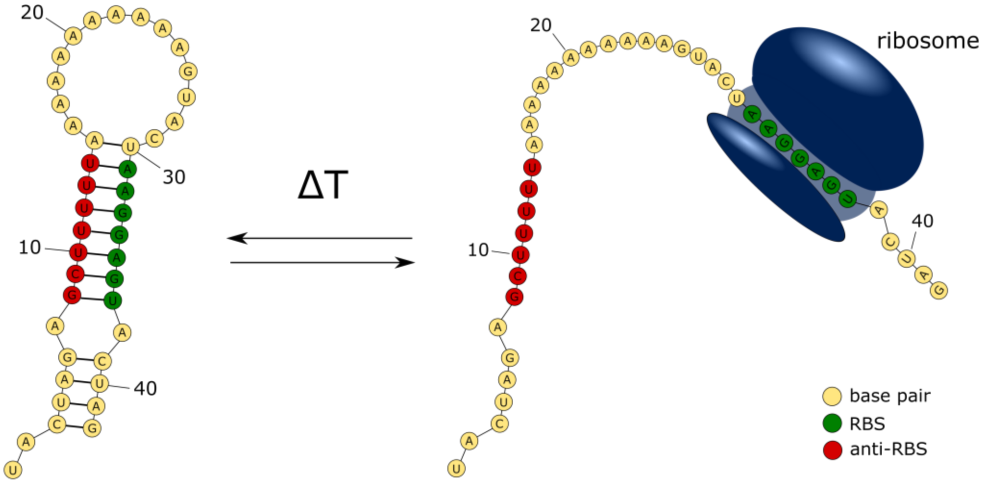

Figure S4: Schematic of temperature-responsive dynamics in the RNA thermometers. The ribosome binding site (RBS) and anti-ribosome binding site are highlighted with different colours.

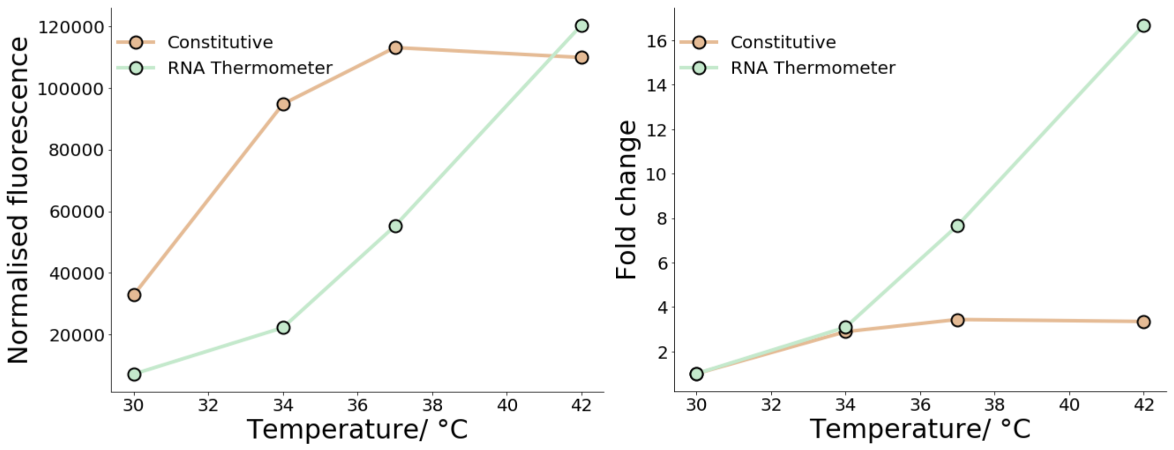

Figure S5: Comparing the RNA thermometers to constitutive bacteria. At temperatures below 42 °C the constitutive bacteria expressed more GFP than the RNA thermometers. However, at 42 °C the RNA thermometers had a larger fluorescent readout. When converted to a fold change the RNA thermometers exhibited much greater switching behaviour than the constitutive bacteria demonstrating that the RNA constructs were working and giving rise to a greater temperature dependence on GFP production than expected from the constitutive strain.

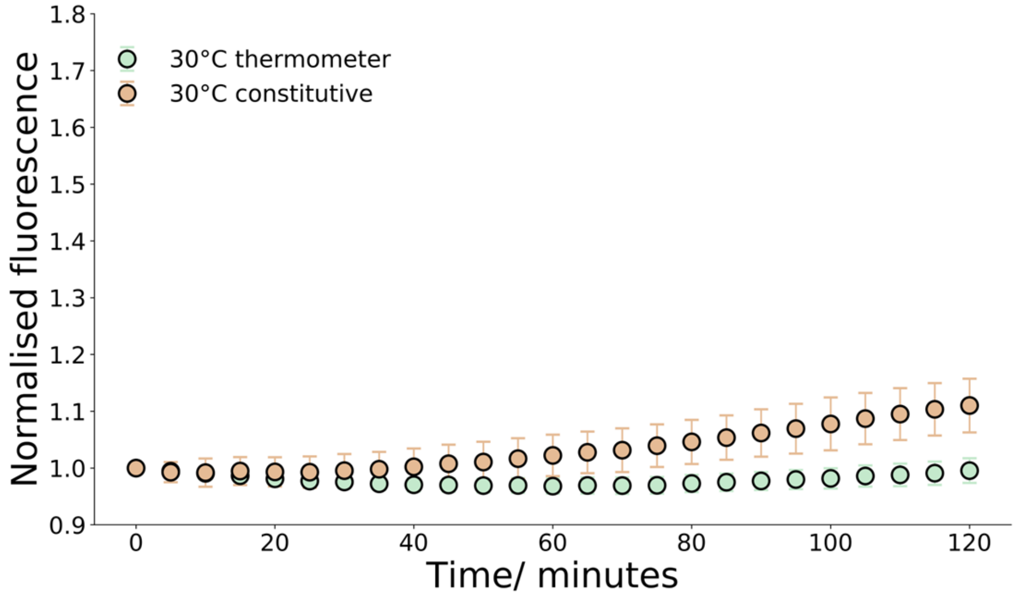

Figure S6: Comparing constitutive and RNA thermometer bacteria within microgels. It can be seen that at 30 °C the constitutive bacteria possessed a more pronounced GFP response, this matches the bulk data in Figure S5 where the constitutive bacteria had a greater fluorescence response than the RNA thermometers at 30 °C. This demonstrates that the properties the bacteria possess in bulk are transferred into the microgels. The error bars indicate 1 standard deviation from n=10 hydrogel beads.

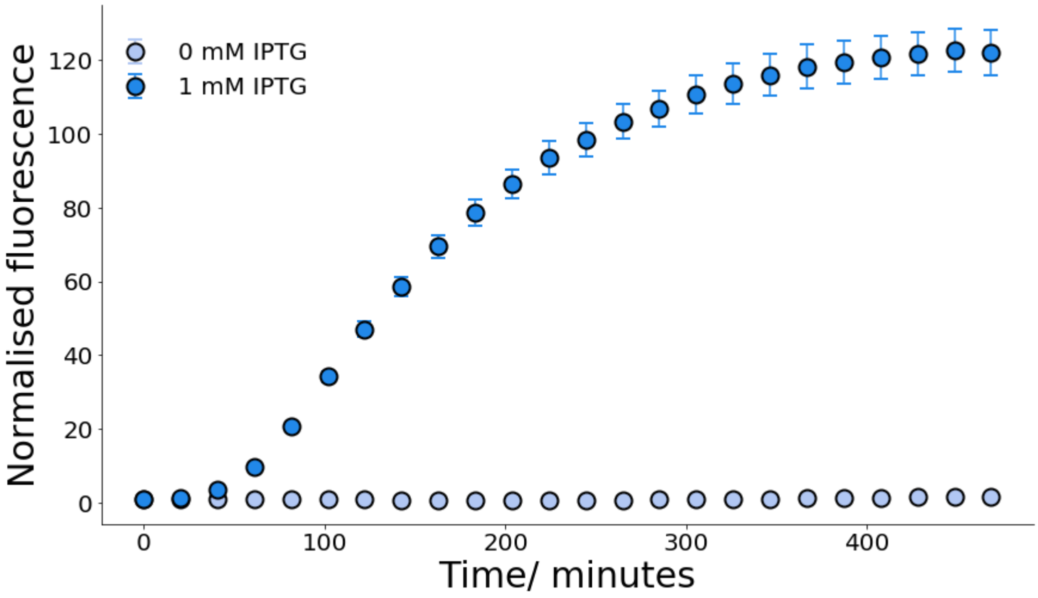

Figure S7: Determining IPTG response in bacteria colonies. Upon incubation with IPTG, the IPTG responsive bacteria showed an increase in fluorescence due to the production of GFP. No response was seen without IPTG demonstrating that the IPTG response works in bulk bacterial colonies. The error bars indicate 1 standard deviation from n=3 bacteria colonies.

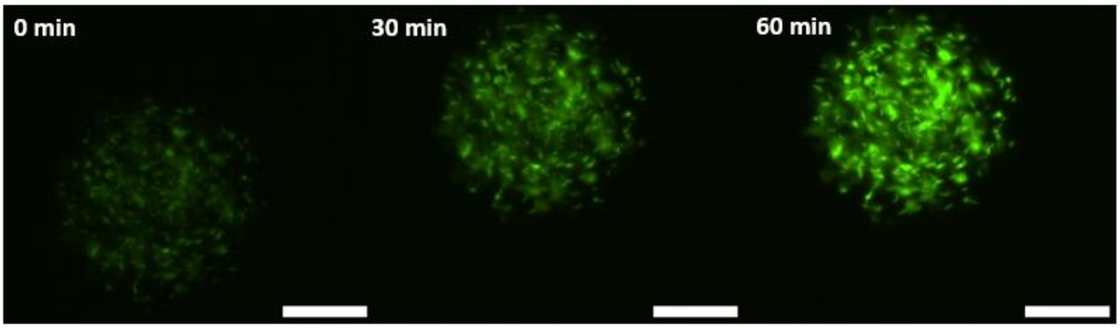

Figure S8: Bacteria response after switching activation conditions. Fluorescence images of bacteria filled microgels that had been activated for 1 hour at 30 °C with 10 mM IPTG in a new solution containing 1 mM IPTG. Another increase in fluorescence was observed over the 1-hour experiment at 30 °C showing that the bacteria filled microgels can respond again to stimuli triggers again after an initial activation. The scale bars are 50 μm.

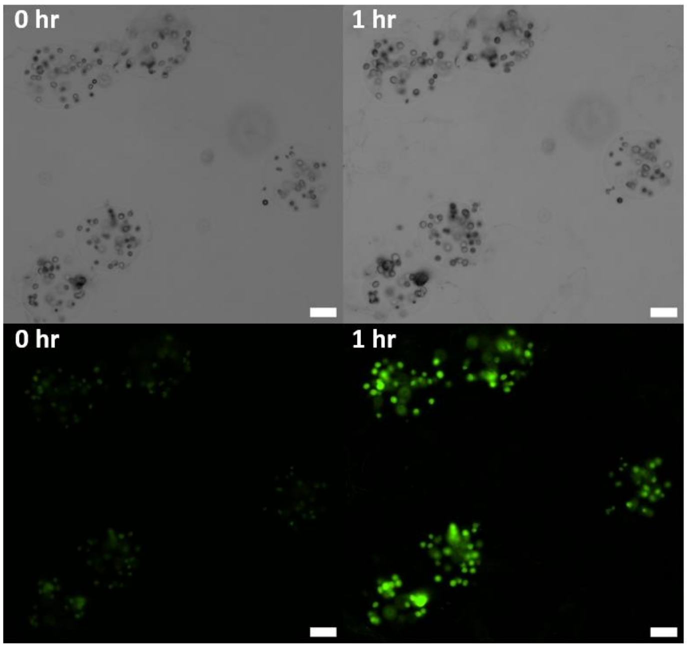

Figure S9: Bacteria response after overnight growth in microgels. Brightfield and fluorescence images of bacteria filled microgels that had been left at room temperature overnight (images on the left). 10 mM of IPTG was then added to the bacteria filled microgels. After 1 hour in 10 mM IPTG (images on the right) it could be seen that the fluorescence of the bacteria structures had increased, demonstrating activity. The scale bars are 50 µm.

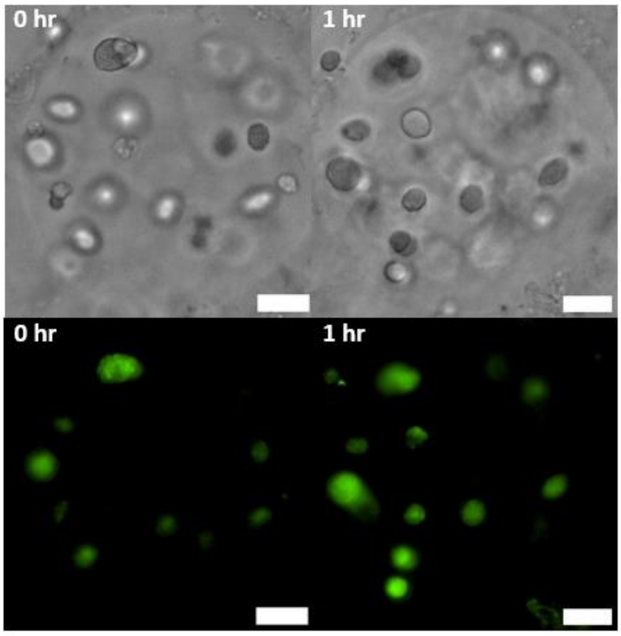

Figure S10: Microgel stability and bacteria activity after 7 days. Brightfield and fluorescence images of a bacteria filled microgel that had been left at room temperature for 7 days (images on the left). 10 mM of IPTG was then added to the bacteria filled microgel. After 1 hour in 10 mM IPTG (images on the right) it could be seen that the fluorescence of the bacteria structures remained consistent, showing limited bacteria activity after 7 days. However, the gel remained intact confirming that the microgels are stable for extended periods of time. The scale bars are 20 µm.

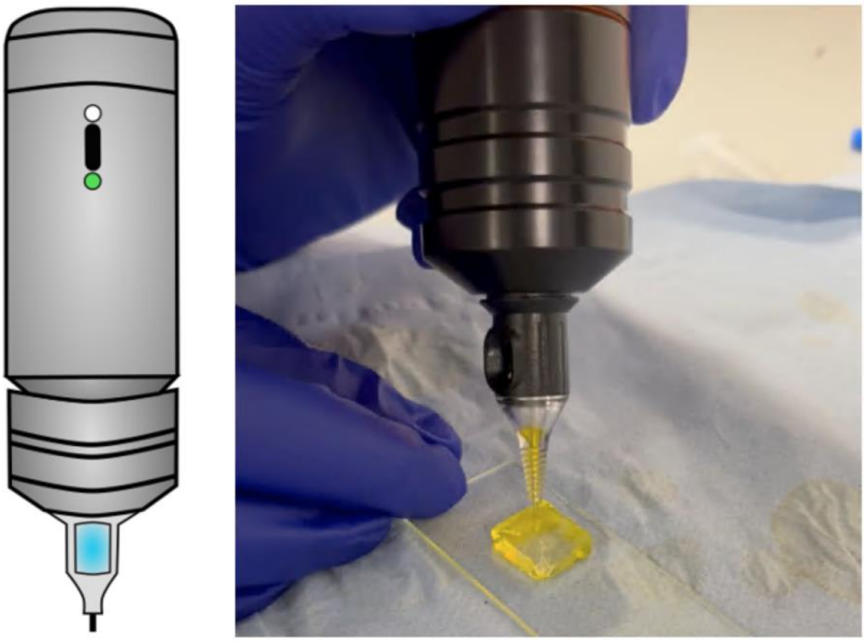

Figure S11: Schematic and photograph of the commercial tattoo gun. An illustration and accompanying photograph of the commercial tattoo gun used. In the photograph the tattoo gun is being utilised to tattoo fluorescent Dextran into the agarose skin mimics (Fig. 3D).

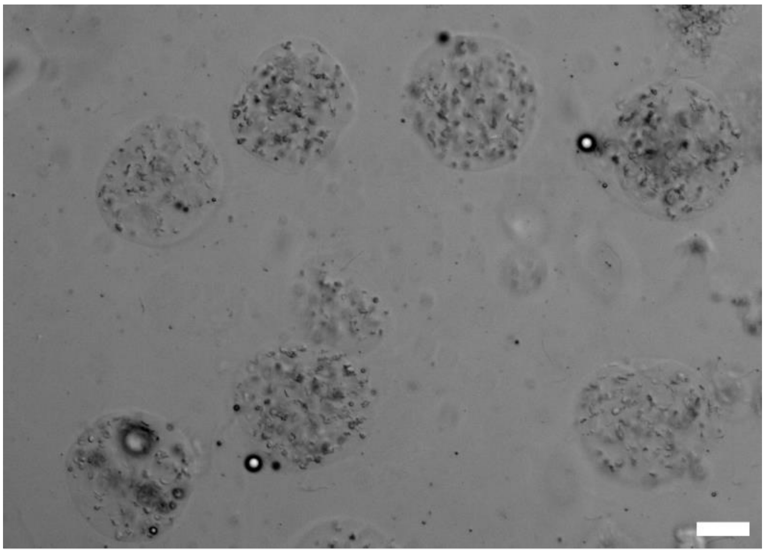

Figure S12: Tattooing of the hydrogel encapsulated bacteria into LB solution. A brightfield image showing that the structure of the hydrogels remains intact upon being passed through the tattoo gun and into fresh LB solution. This demonstrates that tattooing of the hydrogels has no impact upon their structure. The scale bar is 50 μm.

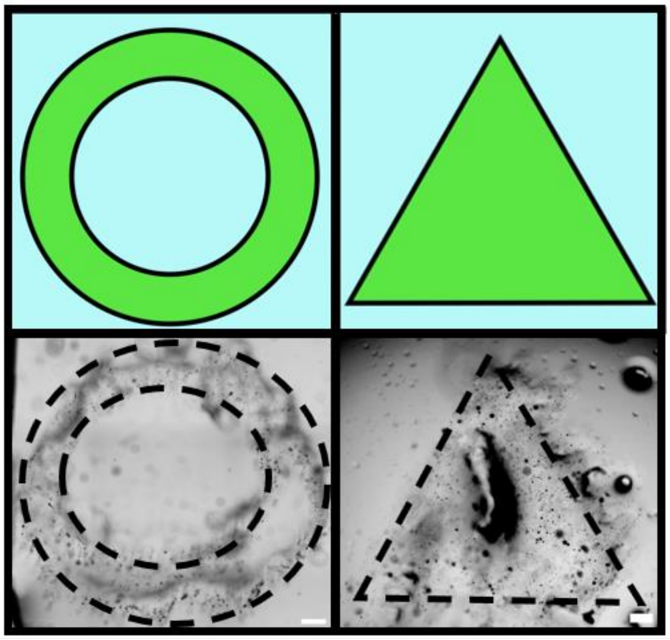

Figure S13: Brightfield images of tattooed shapes with bacteria filled microgels. Schematics of a ring and an infilled triangle tattoo with the corresponding brightfield images below. The dotted lines mark the tattooed region on the agarose skin mimic. A change in texture can be seen where the tattooing has occurred. The scale bar for the ring is 500 µm and 200 µm for the triangle.

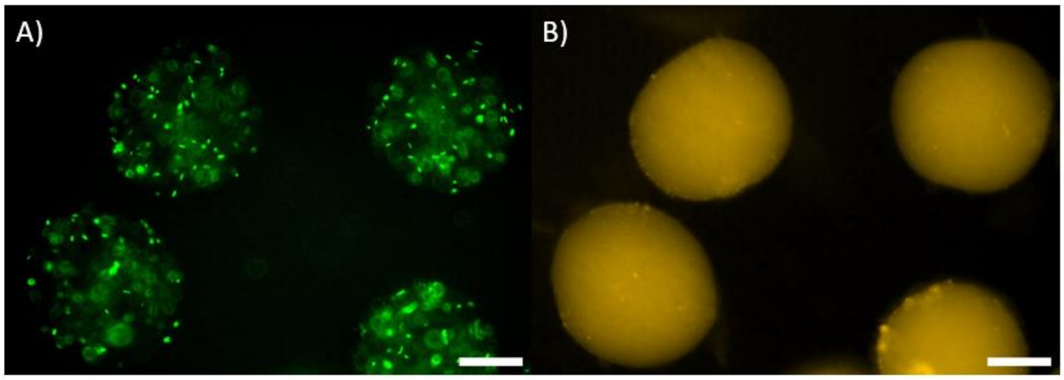

Figure S14: Images of fluorescently labelled microgels with embedded bacteria. A) A fluorescence image showing the localisation of fluorescent signal to the bacteria within fluorescent alginate microgels. **B)** A fluorescence image showing the fluorescence of the fluorescently tagged alginate microgels. These images demonstrate that the fluorescent labelling had no impact upon both bacteria incorporation and observation of the fluorescent signal from the bacteria. The scale bars are 50 µm.

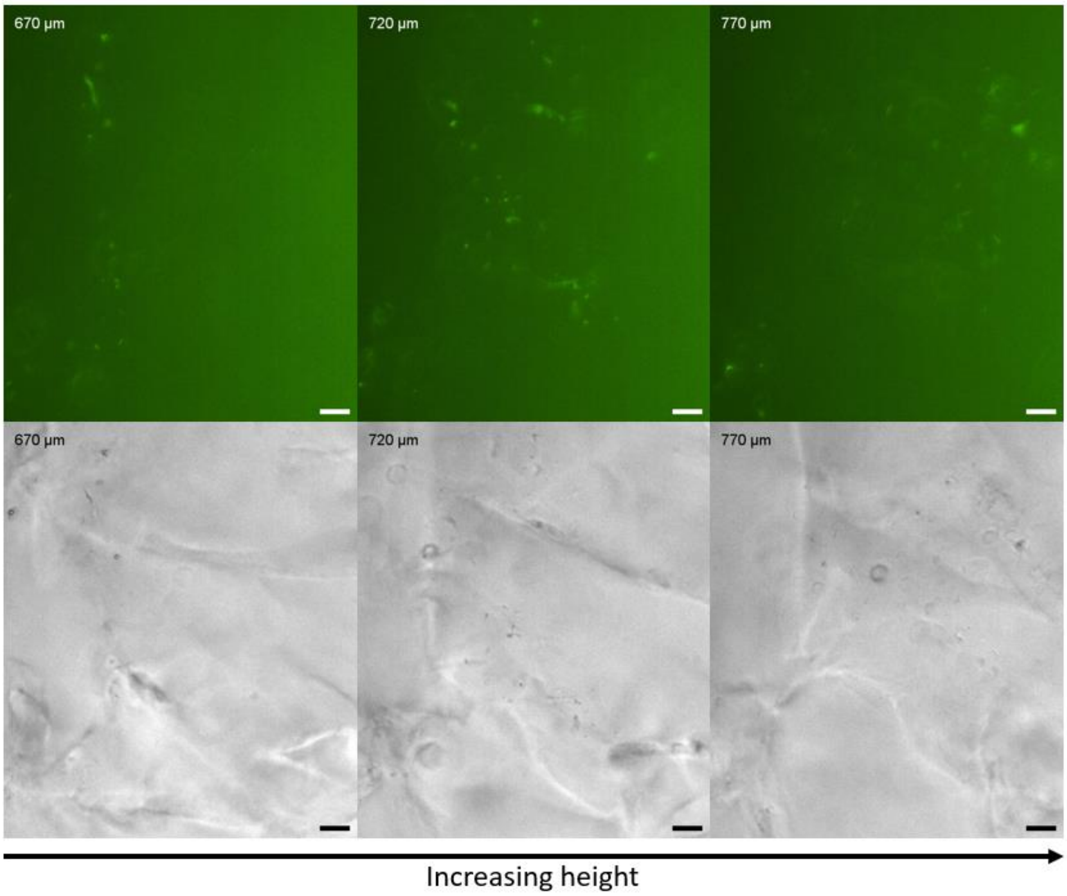

Figure S15: Tattooing unencapsulated bacteria into agarose gels. Fluorescence and accompanying brightfield images showing unencapsulated bacteria tattooed into an agarose skin mimic at a variety of heights. The bacteria can be seen to be dispersed throughout the tattooed region, unprotected from the surrounding environment. The scale bars are 20 µm.

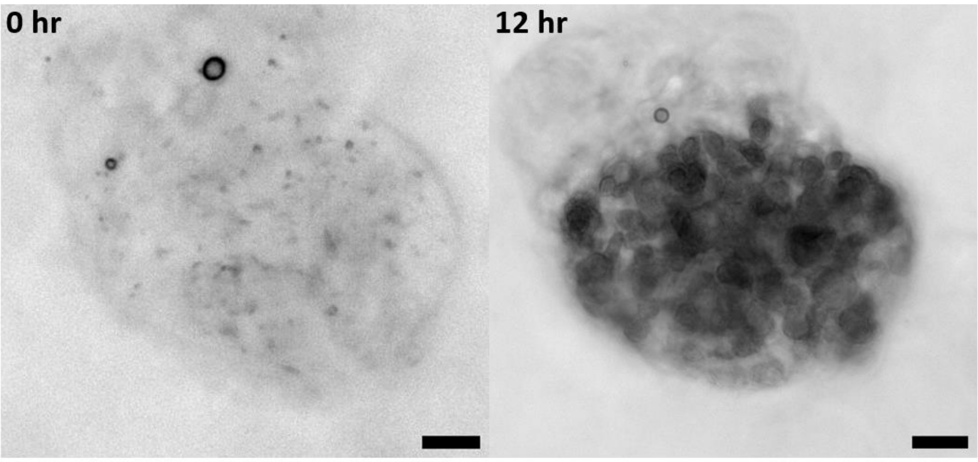

Figure S16: Growth of tattooed bacteria filled microgels in an agarose skin mimic. Brightfield images at 0- and 12-hours showing growth of bacteria within a tattooed microgel. The bacteria growth remained within the tattooed microgels, showing that the bacteria was active and held within the tattooed microgels. The scale bars are 20 µm.

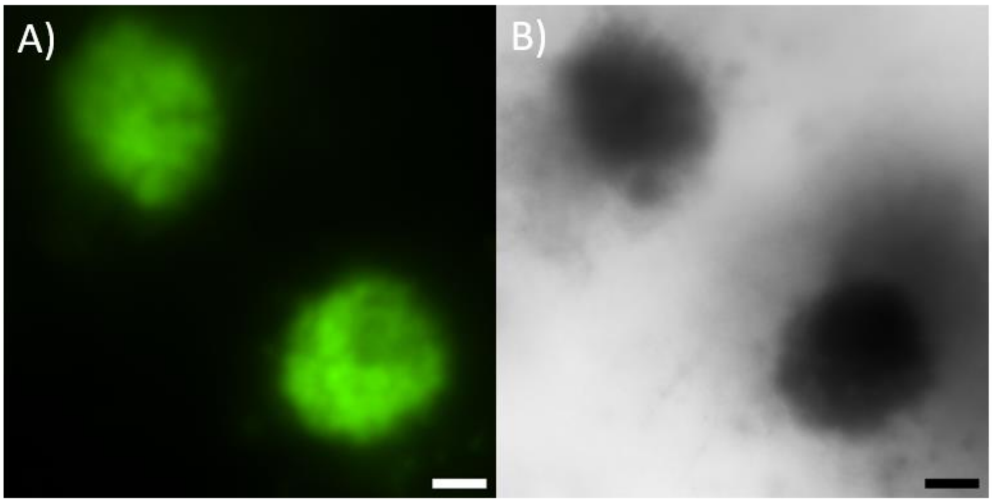

Figure S17: Stability of tattooed bacteria filled microgels in an agarose skin mimic. A fluorescence (A) and brightfield (B) image showing tattooed bacteria filled microgels after 5 days at room temperature within a tattooed agarose gel containing 10 mM IPTG. The fluorescence signal can be seen to be localised to the tattooed microgels. This indicates that the microgel is stable and holds the bacteria microcolonies upon being tattooed for extended periods of time, matching the results in buffer. The scale bars are 50 µm.

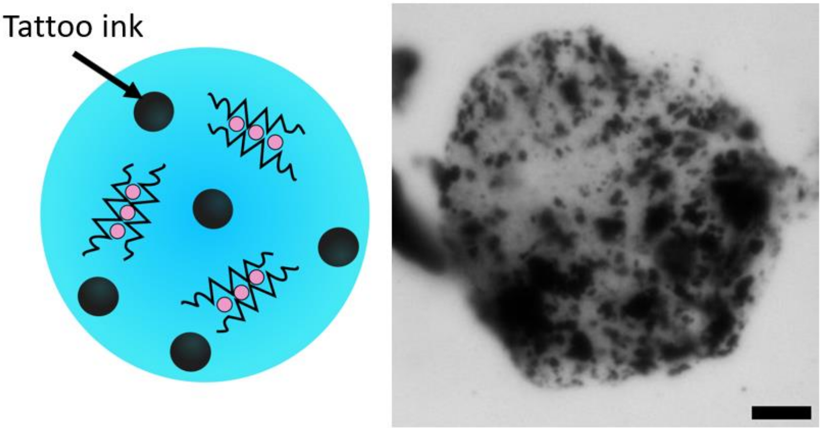

Figure S18: Encapsulation of tattoo ink within the microgels. A schematic and brightfield image demonstrating encapsulation of tattoo ink within the produced microgels. The tattoo ink appears as dark regions within the microgel structure. The scale bar is 20 µm.

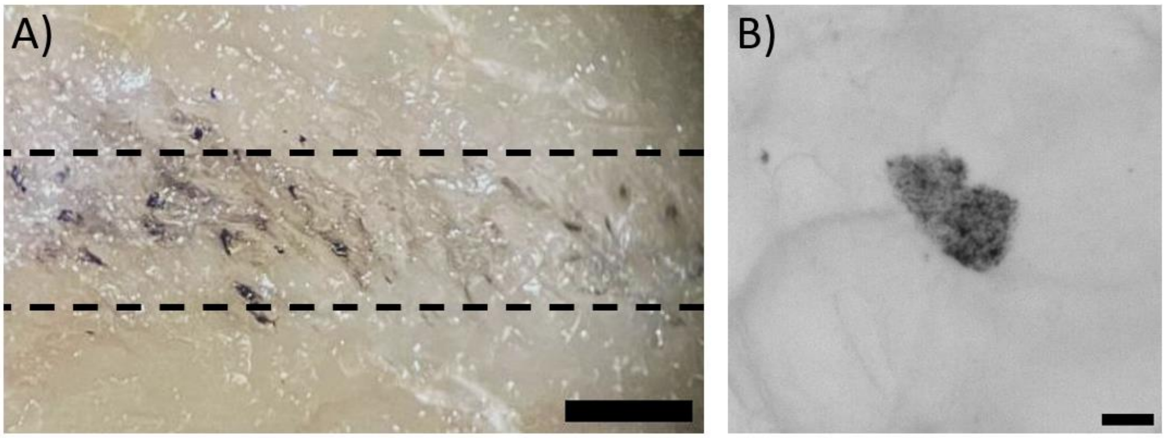

Figure S19: Tattooing tattoo ink filled microgels into porcine skin. A) An image of porcine skin with a region tattooed by the tattoo ink filled microgels (shown by the dashed lines). The tattoo ink filled microgel solution is localised to the tattooed region. The scale bar is 500 µm. **B)** A brightfield image of a tattooed tattoo ink filled microgel in the porcine skin. The tattoo ink remains localised in a microgel section, providing evidence that the microgels can be tattooed in porcine skin. The scale bar is 20 µm.

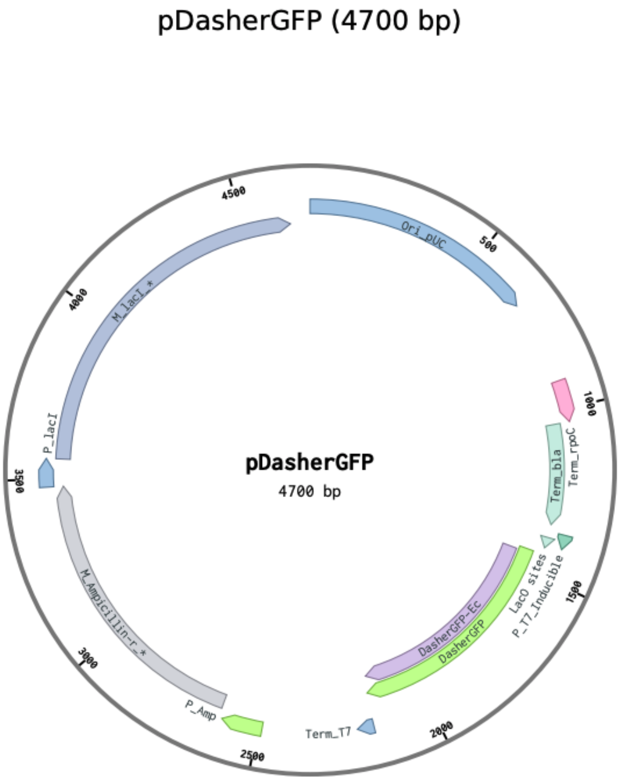

Figure S20: Plasmid map of the IPTG-inducible Dasher GFP.

BBa-J23118b RNAT3-2 dasherGFP (3032 bp)

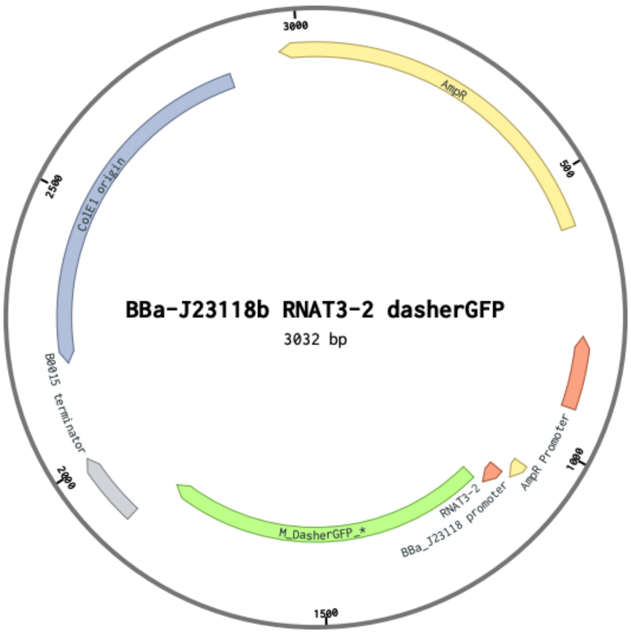

Figure S21: Plasmid map of the RNA thermometer regulator for Dasher GFP.

SI video descriptions

Video S1- A timelapse of GFP production at 40 °C in a microgel containing bacteria controlled by an RNA thermometer. The scale bar is 20 μm.

Video S2- A timelapse of GFP production in a microgel containing bacteria induced by 0.1 mM IPTG. The scale bar is 20 μm.

Video S3- A brightfield timelapse of bacteria growth in the microgels at room temperature over 12 hours. The scale bar is 20 μm.

Video S4- A upwards Z stack of tattooed microgels throughout a section of the agarose skin mimic. The scale bar is 50 μm.

Video S5- A timelapse of GFP production from the bacteria in a tattooed microgel induced by 1 mM IPTG. The scale bar is 20 μm.
